# Energy efficient virtual machines placement in cloud datacenters using genetic algorithm and adaptive thresholds

**DOI:** 10.1371/journal.pone.0296399

**Published:** 2024-01-02

**Authors:** Abdullah Alourani, Aqsa Khalid, Muhammad Tahir, Muhammad Sardaraz

**Affiliations:** 1 Department of Management Information Systems and Production Management, College of Business and Economics, Qassim University, Buraidah, Saudi Arabia; 2 Department of Computer Science, COMSATS University Islamabad, Attock Campus, Attock, Pakistan; Jazan University Faculty of Computer Science, SAUDI ARABIA

## Abstract

Cloud computing platform provides on-demand IT services to users and advanced the technology. The purpose of virtualization is to improve the utilization of resources and reduce power consumption. Energy consumption is a major issue faced by data centers management. Virtual machine placement is an effective technique used for this purpose. Different algorithms have been proposed for virtual machine placement in cloud environments. These algorithms have considered different parameters. It is obvious that improving one parameter affects other parameters. There is still a need to reduce energy consumption in cloud data centers. Data centers need solutions that reduce energy consumption without affecting other parameters. There is a need to device solutions to effectively utilize cloud resources and reduce energy consumption. In this article, we present an algorithm for Virtual Machines (VMs) placement in cloud computing. The algorithm uses adaptive thresholding to identify over utilized and underutilized hosts to reduce energy consumption and Service Level Agreement (SLA) violations. The algorithm is validated with simulations and comparative results are presented.

## Introduction

Cloud computing provides on-demand delivery of computing services, storage, and applications. In cloud computing, resources are only a click away which saves time. Cloud services are constrained by parameters such as Quality of Service (QoS), user deadlines, user budget, execution time, energy consumption Service Level Agreements (SLAs) and many more [1, 2]. Different techniques have been opted to balance these parameters. Virtual Machines (VMs) placement is one such technique. It is the process of finding suitable hosts for different VMs. It is an important operation to control the assignment of VMs to physical machines with the changes in application load. It is necessary to maintain the utilization record of the host to reduce energy consumption [3]. Each VM request is individually characterized by four parameters i.e., CPU, memory, disk, and bandwidth. Resource allocation algorithms are used to accept VM requests as many as possible. Different types of allocation strategies work on the basis of allocation policy to maintain the number of VMs. We can virtualize the hardware, servers, and operating system to minimize energy consumption and cost.

**Fig 1:**
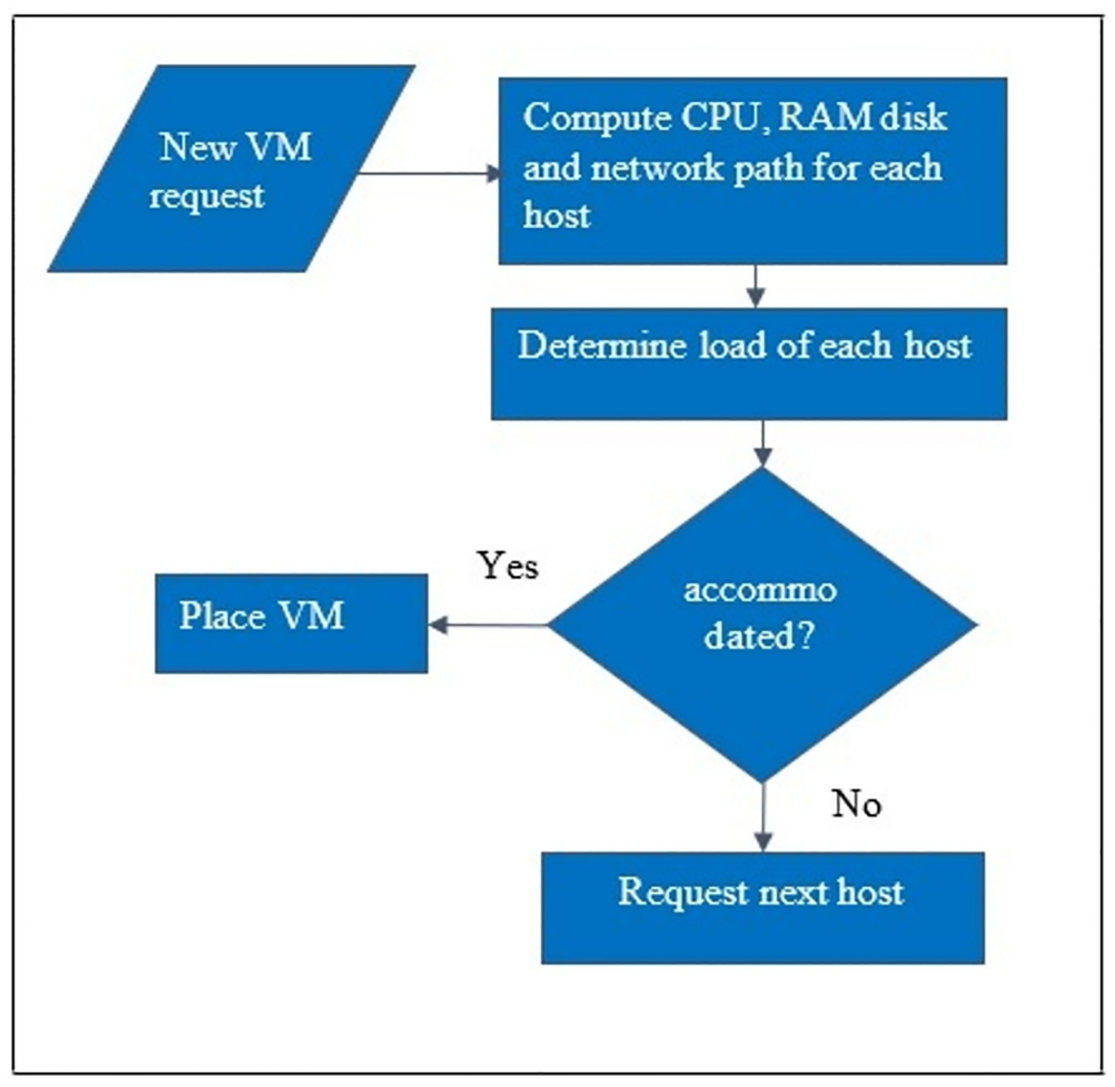
Flow of VM placement algorithms.

Energy consumption in cloud data centers increases with the number of users and services. Research has been carried out to detect overload or underload hosts at runtime to reduce the energy consumption in cloud data centers using VM placement algorithms [4]. The purpose is to effectively place VMs in data centers to ensure the QoS and reduce energy consumption. The objective of the VM placement algorithms is to reduce energy consumption i.e., to reduce the sum of energy used by the computation, processing, transfer, and storage [5]. The procedure of VM placement is shown in Fig 1. Where the scheduler determines the load of a host. If the host can accommodate VMs, the placement takes place otherwise, the next available host is checked.

In cloud data centers, energy consumption can be reduced by increasing the number of VMs and decreasing physical machines [6]. Activation of the active and idle modes of VMs also decreases energy consumption and enhances resource utilization [7]. Sorting of tasks according to the schedule of processing needs to avoid overload or underload hosts in data centers and reduce energy consumption. Mapping of the task from one VM to other VMs and the assignment of the physical machine to VMs also consumes energy and needs to be addressed [8].

In this article, we present a novel algorithm for VM placement in cloud computing environments. The algorithm is based on Genetic Algorithm (GA) with adaptive threshold. Adaptive thresholds are used to detect over-utilized and underutilized hosts in cloud data centers. Setting static upper and lower thresholds may lead to imbalance load and thus cause more energy consumption and low QoS. The proposed algorithm uses an intermediate value as the average of the upper and lower thresholds to identify hosts with utilization near the lower threshold. The resources of these hosts are allocated to VMs for better resource utilization. Furthermore, GA is used to optimize the allocation process. The objectives are to reduce energy consumption with low SLA violations for better QoS. The algorithm is validated by experiments with different configurations and comparative results are presented. The rest of the paper is organized as follows. The literature review section presents a detailed discussion on state of the art methods, followed by materials and methods section that covers the proposed method in detail. The results and discussion section presents the experimental evaluation followed by the conclusion section.

## Literature review

The applications of cloud computing are diversified and cover a large number of domains including health, education, business, supply chain, and many more [9–11]. Increase in applications leads to massive amounts of data and processing. The processing is achieved using virtualization i.e., VM placement. VM placement is a critical factor in resource utilization and power efficiency in cloud computing. The designed algorithms must consider the dynamic nature of the workload and live VMs migration. Several evolutionary and non-evolutionary algorithms are used for VM placement in cloud computing. Algorithms for VM placement may be static or dynamic. In static algorithms, decisions are not reviewed later whereas in dynamic algorithms, the assignment of VMs to host changes during execution [12]. The basic task of a scheduler is to achieve the desired objectives with an efficient VMs schedule [13]. Another issue faced by cloud customers is the security threat, where private information of the user is extracted from VMs located on some server [14]. Dynamic allocation of VM can also improve the allocation policy so that it is difficult for the attacker to locate the targets [15]. There exist reviews on VM placement and scheduling in cloud computing. In this section, we discuss some of these techniques that are closer to the work of this article. Readers are referred to the reviews [5–8, 16, 17] for details of different methods.

An algorithm for VM placement and load assignment is proposed in [18]. The algorithm is based on a mathematical model to reduce hardware requirements for VMs. The algorithm targets workload in multi-application environments by considering the heterogeneous latency requirements of different applications. Numerical results are shown to validate the proposed algorithm. To resolve the issue of energy consumption in cloud data centers, authors in [19] proposed a hybrid algorithm. The algorithm is based on Random Forest (RF) and GA. The objectives of the algorithm are to reduce energy consumption with better load balance. GA is used to generate the training dataset used to train RF model. Comparative experimental results are presented using real-time workload traces. The results analysis show that the proposed model performs better than the existing compared models. An algorithm based on utility functions for VMs placement is proposed in [12]. The goals of the algorithm are to optimize energy consumption and SLA violations (SLAVs). The algorithm uses fewer VM migrations and hosts shut down to achieve the desired goals. Simulation results using CloudSim are shown to validate the proposed algorithm. A distributed parallel genetic algorithm for VMs placement in cloud environment is presented in [20]. The algorithm runs on different hosts in parallel and distributed fashion. The solutions obtained from this stage are used as inputs to the next stage as the initial population. The algorithm searches for an optimal solution in the second stage. The objectives of the algorithm are to ensure QoS and low energy consumption. CloudSim simulator is used to validate the performance of the proposed algorithm. The proposed model in [21] is based on a comprehensive system model that shaped a problem as a constrained multi objective optimization. It includes a novel thermal aware VM placement strategy that considers cost increment, energy consumption, migration cost, and calculation of heat that circulates around the server racks. Placement decisions are taken with the help of Multi Objective Algorithm based on Path Finder Algorithm and Genetic Algorithm (MOPFGA). The combined algorithms are enhanced by Opposition Based Learning (OBL) to avoid the local optimum. Improvements in different parameters are claimed after experimental analysis.

Authors in [22] proposed a Multi-resource Collaborative Optimization Control (MCOC) mechanism for the migration of VMs to address the problems of imbalance workload, resource wastage, and negative impact on QoS. These problems come from unbalanced resource utilization of physical machines in cloud data centers. For adaptive estimation of the probability that the functional machines are in the multi-resource utilization balance status, the Gaussian model is used. This study proposed an effective selection algorithm for VM migration between the source and destination host. It also includes adaptive Gaussian Model-based VM placement (AGM-VMP) algorithm and VM consolidation (AGM-VMC) method. The results show that the AGM-VMC method is an effective method for improving resource utilization and achieve load balance. It also reduces energy consumption in data centers. In the comparison of the same method with the statistic and heuristic methods, intelligent algorithms can normally obtain an appropriate optimal solution. Therefore, these methods are more efficient in dealing with complex workloads that are not balanced in VM placement environment. However, this algorithm needs iterations in evaluation which causes long execution time and less efficiency. Authors in [23] proposed an algorithm for reliable VM placement and routing in cloud computing. The algorithm targets the availability of VMs in response to consumers’ requests. A specific number of hosts and VMs are ensured to be available such that communication delays and the number of VMs to host are not violated. The algorithm uses an exact Integer Nonlinear Program (INLP) to solve the problem. The algorithm also studies the problem of reliable routing. The target is to ensure the availability and keep the delay under a threshold. The algorithm is validated with the experimental results. Authors in [24] considered the co-location attack problem that occurs in cloud Infrastructure as a Service (IaaS) layer as the result of VM placement strategies. The study proposed an online secured optimization VM placement that ensures a high level of security by minimizing the use of minimum physical servers. The results show the efficiency and correctness of the system. The method ensures security by the cloud provider for its customers and is very useful in practice. The core component of the proposed strategy is the statistical security inference method in which evaluation is done. It depends on the new statistical symbol assignment, which allows the provider to specify a confidence level and an upper-bound deviation of security in addition to the security parameters. This approach was validated empirically over multiple configurations and kept errors under control. Authors in [25] divide the problem of VMs placement into sub-problems such as the detection of overutilized and under-utilized hosts, VM migration, and selection policies. Different heuristics have been adopted and evaluated in terms of energy consumption, SLAVs, VM migration, etc. Another algorithm for VMs placement in cloud environments is proposed in [26]. To ensure the reliability of cloud services, a redundant VM strategy is used. The solution consists of three algorithms. The first algorithm is used to select hosts for VMs. The second algorithm is used to place the VMs on the selected host and the third algorithm is used to dynamically assign tasks to VMs which is based on finding maximum weight matching in bipartite graphs. The objective of the algorithm is to ensure reliability in cloud services.

Maintaining the efficiency of energy is a critical issue in data center management. The placement of VMs in a good way increases the efficiency of energy. The proposed method in [27] worked on accelerated GA for energy efficiency in VM placement. This method simplifies the calculations in GA using a new fitness function. Simulation results demonstrate that the accelerated GA produces better results as compared to the standard GA on small, medium, and large-scale data centers. More physical machines can be switched off as compared to standard GA to save more energy. The results of updated GA with the new fitness function derives an 8% energy saving and a 66% reduction in execution time as compared to the standard GA. In reference [28], researchers proposed a model to solve the problem of energy consumption and replacement time. The proposed performs integration on underutilized servers for energy saving. Afterward, an improved Long Short-Term Memory (LSTM) model is utilized that reduces latency based on historical data. Results show that the improved learning model can reduce the placement latency and can save more energy. Authors in [29] proposed an improved algorithm for VM placement which is based on Ant Colony Optimization (ACO) which contains effective modern processor technologies and parallelization techniques. The method is faster than the compared method. A two-phase optimization algorithm for VM placement in cloud computing is presented in [16]. The algorithm combines the advantages of online and offline formulation in dynamic environments. The objectives are service elasticity and overbooking of physical resources. The algorithm targets power consumption, revenue, resource utilization, and reconfiguration time. The two phases include the reconfiguration of VM migration and handling the requests during the reconfiguration phase. Experimental evaluation is presented to validate the proposed algorithm.

Another algorithm that minimizes the number of physical machines for VM placement is proposed in [30]. The algorithm is based on non-linear programming to formulate the problem. In addition to server resources, additional constraints are added to guarantee the placement. Simulation results are shown to validate the proposed algorithm. Another algorithm that targets cloud security in VM placement is proposed in [31]. The algorithm uses grouping-based placement strategy to achieve the desired goal. The approach is analyzed both theoretically and with simulation results to show the viability of the proposed solution. It is shown that the performance slightly decreases with implanting security based VM placement algorithm. An energy-aware ant colony system for VM placement in cloud computing is proposed in [32]. The algorithm minimizes the number of physical servers to identify under utilized servers and reduce energy consumption. The algorithm uses ant colony system coupled with order exchange and migration local search to solve the problem. Experimental results are shown to validate the performance of the proposed algorithm. Another algorithm for VMs placement is presented in [33]. The algorithm is based on Ant Colony Optimization (ACO) and targets power consumption and VM performance as bi-objective optimization problem. The algorithm builds a power model based on CPU utilization and power consumption. VM performance is dealt with by the VM performance model. The performance of the algorithm is validated with the experimental results. Another algorithm for energy-efficient VM placement is presented in [34]. The algorithm is based on the decrease and conquers GA. The algorithm decreases the problem size and the number of VM migrations. The algorithm is compared with other techniques to validate the performance. Cloud computing is also being challenged by the correlation between energy consumption and QoS. The challenges include resource utilization and energy consumption. Under utilization or over utilization ultimately leads to degrading QoS. To overcome these problems, a technique based on adaptive thresholds is proposed in [35]. This technique is based on a three-way decision in VMs migration. In the first step, the algorithm evaluates the load of hosts according to the utilization of each host. The algorithm determines dynamic thresholds to identify under and over-utilized hosts. Based on the values of thresholds, the algorithm categorizes the hosts as under-utilized, normal, or over-utilized. Placement takes place according to the status of each host i.e., normal nodes do nothing and for the other two nodes, VMs will be determined by the ATM to migrate and optimize the target host choice based on the transmission overhead.

Many solutions exist to solve the problem of energy consumption in cloud data centers. Different researchers have targeted different parameters. The solutions also consist of optimization techniques with one or more objectives. VM placement and load balancing are used to solve this problem. The parameters are related to each other i.e., improving one parameter affects the other parameters. Thresholding is used to measure the load of hosts to avoid over and under-utilization of the resources. This results in better resource utilization, low energy consumption, and better QoS. Usually lower and upper thresholds are used to detect overutilized and underutilized hosts. In some cases, the utilization remains closer to the lower threshold, however, the lower threshold does not detect such utilization. This scenario leads to inefficient resource utilization despite using thresholds. The solutions must also take care of the trade-off between different parameters.

## Materials and methods

This section presents the proposed algorithm in detail. First, the cloud computing model and adaptive thresholding are discussed followed by energy consumption, SLA violations, and GA. The proposed method is presented at the end of the section.

### Cloud model

In cloud data centers, physical infrastructure is provided with Physical Machines (PMs) or hosts. Each data center consists of a set of hosts (*H*_1_, *H*_2_, …, *Hn*). The hosts are virtualized to create VMs to execute users’ tasks. A host *H*_*i*_ running in the data center is considered as active host. Each active host is represented as *H*_*i*_ = ((*f*_*i*_, *v*_*i*_), *C*_*i*_, *M*_*i*_, *S*_*i*_) where *f*_*i*_ and *v*_*i*_ are the pairs of frequency and voltage whereas *C*_*i*_, *M*_*i*_ and *S*_*i*_ represent computation, memory and storage capacity respectively [36]. After virtualization, the resources are allocated to VMs. The allocation of resources to *VM*_*j*_ can be represented as $V_{j}=\left(V_{j}^{c},V_{j}^{m},V_{j}^{s}\right)$ where $V_{j}=\left(V_{j}^{c},V_{j}^{m},V_{j}^{s}\right)$ represent computation, memory and storage demand of *VM*_*j*_. Eqs 1 to 3 show allocated resources of the host to n VMs [35, 37].

$$\begin{array}{c} p_{i}=\sum_{i=1}^{n}\;MIPS_{j} \end{array}$$ (1)

$$\begin{array}{c} m_{i}=\sum_{i=1}^{n}\;mem_{j} \end{array}$$ (2)

$$\begin{array}{c} d_{i}=\sum_{i=1}^{n}disk_{j} \end{array}$$ (3)

Where *n* is the number of VMs allocated to host *H*_*i*_.

The weighted sum of the resource utilization of a host is calculated with Eq 4. Where *α*, *β* and *γ* are the weight factors of each resource. Based on load of a host, the host can be categorized as over utilized, under utilized, or normal [35].

$$\begin{array}{c} U=\alpha \frac{\sum_{i=1}^{n}c_{i}}{C}+\beta \frac{\sum_{i=1}^{n}m_{i}}{M}+\gamma \frac{\sum_{i=1}^{n}s_{i}}{S} \end{array}$$ (4)

Thresholds *T*_*l*_ and *T*_*u*_ are used to adaptively adjust the load of a host. Thresholds are calculated with Eqs 5 and 6 An intermediate value *m* is also calculated to further check the load of normal hosts. The value is calculated as the average of upper and lower thresholds as shown in Eq 7.

$$\begin{array}{c} T_{l}=U_{s}-\sigma \end{array}$$ (5)

$$\begin{array}{c} T_{u}=O_{s}+\sigma \end{array}$$ (6)

$$\begin{array}{c} m=\frac{T_{l}+T_{u}}{2} \end{array}$$ (7)

In Eqs 5 and 6 *σ* refers to the standard deviation of the load in the cluster. The standard deviation is calculated with Eq 8. Whereas *U*_*s*_ and *O*_*s*_ represent the evaluation values of the cluster underload and overload status calculated with Eqs 9 and 10 respectively.

$$\begin{array}{c} \sigma =\sqrt{\frac{\sum_{i=1}^{n}{\left(L_{i}-\bar{L}\right)}^{2}}{n}} \end{array}$$ (8)

Where *L*_*i*_ is to the load of *i*_*th*_, $\bar{L}$ is the average load of all hosts and *n* is the number of hosts.

$$\begin{array}{c} U_{s}=\bar{L}+m_{1} \end{array}$$ (9)

$$\begin{array}{c} O_{s}=\bar{L}+m_{2} \end{array}$$ (10)

Where $\bar{L}$ is the average load of all hosts in the cluster, *m*_1_ and *m*_2_ are the boundary functions calculated with Eqs 11 and 12 respectively.

$$\begin{array}{c} m_{1}=\{\begin{array}{cc} \mu (\bar{L}\left(1-optimal\right), & \text{if}\;(1-optimal)\leq \bar{L}.\\ 0, & \text{otherwise}. \end{array} \end{array}$$ (11)

$$\begin{array}{c} m_{2}=\{\begin{array}{cc} \omega (optimal-\bar{L}), & \text{if}\;(optimal)\geq \;\bar{L}.\\ 0, & \text{otherwise}. \end{array} \end{array}$$ (12)

Where 1 − *optimal* is the complementary point of the tradeoff between performance and energy consumption, *μ* and *ω* are the boundary factors. In Eqs 11 and 12, *optimal*, *μ* and *ω* are calculated with Eqs 12 through 15 respectively.

$$\begin{array}{c} optimal=\{\begin{array}{cc} (1-\bar{L}), & \text{if}\;\bar{L}\leq \text{0.5}.\\ 0.5, & \text{otherwise}. \end{array} \end{array}$$ (13)

$$\begin{array}{c} \omega =\{\begin{array}{cc} (1-\sigma ), & \text{if}\;\sigma \leq \text{0.5}.\\ 0.4, & \text{otherwise}. \end{array} \end{array}$$ (14)

$$\begin{array}{c} \mu =\frac{\omega }{2} \end{array}$$ (15)

### Energy consumption and SLA violations

In cloud data centers, several factors lead to energy consumption including hosts, networks, and cooling systems. Hosts are the main cause of energy consumption [25]. Hosts consume energy through computation, memory, and storage, etc. The energy consumption of a host can be either static or dynamic. Static mode refers to the idle state of the host where approximately 70% of the energy is consumed when the host is running with peak CPU utilization [36]. Dynamic energy consumption refers to energy consumption by different utilization levels of the CPU. The energy consumption model used in this paper is shown in Eq 16.

$$\begin{array}{c} E_{c}=E_{idle}+\left(E_{max}-E_{idle}\right)\times U \end{array}$$ (16)

Where *E*_*max*_ is the energy consumed when the host is at peak utilization, k is the static energy consumption and U is the resource utilization. Utilization is calculated with Eq 4 [35].

SLA violations depend upon multiple parameters. Host utilization is one of the parameters. Overutilization of underutilization of the host leads to VMs migrations that in turn lead to SLA violations. The target is to reduce the overall SLA violations as shown in Eq 17 [12].

$$\begin{array}{c} overallSLAVs=\frac{totalRequestedMIPs-totalAllocatedMIPs}{totalRequestedMIPs} \end{array}$$ (17)

Where totalRequestedMIPs are the sum of MIPs of requested by all VMs and the totalAllocatedMIPs are the MIPs allocated by a host to all VMs. The number of VMs migrations is another factor of SLA violations. Large number of migrations lead to poor performance and too few migrations lead to inappropriate placement. There should be a balanced number of migrations to handle the tradeoff. Host shutdowns also lead to SLA violations. Too frequent switching of hosts might lead to failure in long run [38].

### Genetic algorithm

GA is a class of evolutionary algorithms to solve computationally intensive problems. Phases in GA include the selection of the initial population, evaluation of the fitness, crossover, and mutation. Steps are repeated until no better solution is found or a predefined termination criterion is met. The structure of chromosomes and genes is defined according to the characteristics of a particular problem [39]. GA follows the genetic process to produce new generations. The first genetic operator is the selection that selects the chromosomes for the population. After selecting the initial population, crossover and mutation operators are used to generate new generations. Each chromosome is evaluated and the fittest solutions are finally used. The fittest solutions are selected according to the fitness function [40]. The fitness function used in this article is shown in Eq 18 which is based on the maximum and minimum values of energy consumption and SLA violations.

$$\begin{array}{c} Fitness=w\frac{max^{ec}-ec}{max^{ec}-min^{ec}}+w\frac{max^{SLAVs}-SLAVs}{max^{SLAVs}-min^{SLAVs}} \end{array}$$ (18)

### Proposed algorithm

The cloud model considered in this article consists of *N* heterogeneous hosts with limited resource capabilities. The users’ requests are handled by creating VMs and assigning VMs to hosts. The target is the dynamic allocation of VMs to hosts in such a way that overall energy consumption and SLA violations are reduced. The problem is considered as dynamic allocation which in turn leads to an optimization problem. The proposed algorithm uses GA for optimization purpose. The utilization of hosts and VMs strongly influences the performance of cloud computing. Energy consumption and SLA violation are also influenced by the utilization of resources. The proposed algorithm targets the utilization of hosts and VMs to reduce energy consumption and SLA violations.

In the first phase, the algorithm uses the adoptive thresholds [35] to identify over utilized and under utilized hosts. The algorithm defines two thresholds for host utilization. The lower threshold is used to identify under-utilized hosts whereas the upper threshold is used to identify the over-utilized hosts. Normal hosts i.e. host with normal utilization lies between the two thresholds. In addition to the two thresholds defined, we have adopted a new value i.e. the average of the lower and upper thresholds. This value is used to identify those hosts which are considered normal hosts, but the load is near the lower threshold. This causes inefficient utilization of resources as the algorithm will not assign any more load to this host. The new value identifies these hosts and assigns VMs to the hosts for efficient utilization. The value can be calculated as the average of the lower and upper thresholds as shown in Eq 7. For example, if the lower threshold is 30 and the upper threshold is 90 the algorithm finds a host with 40 percent utilization. The host is considered with the normal load but if no more VMs are assigned, the host resources will be wasted. The new value, in this case, is 60. The proposed technique checks the load of the host. If the load is less than the middle value, the host is considered for VMs assignment. The assignment is conditional i.e. the new load does not cause the host to be overutilized (lines 27–40 of Algorithm 1). The algorithm checks if any assignment causes violations. For this purpose, over-utilized hosts are identified at the time of resource allocation. The algorithm calculates the total resources needed for VMs and the available resources of hosts. If the demand for resources does not cause over-utilization of the host, the algorithm proceeds with the assignment otherwise VMs are assigned to another host with under-utilization. Authors in [12] use this technique to calculate violation cost whereas, in the proposed algorithm, the technique is used to calculate the load during the assignment process. If a host is identified as over-utilized, the VMs are identified for migration (lines 7–10 of Algorithm 1). This step includes the identification of the target host for VMs migration, which is selected based on the current utilization. If a host is underutilized, first it is checked if VMs in the waiting list can be accommodated in this host. If VMs are accommodated, the assignment will take place according to the upper threshold otherwise VMs from the host are added to the migration list, and the host is put into sleep mode (lines 11–26 of Algorithm 1).

**Algorithm 1** Pseudo code of the proposed algorithm

1: **Input**: hostsList

2: **Output**: hosts load

3: calculate *T*_*u*_, *T*_*l*_ and *m*

4: declare migration list *M*_*l*_

5: **for** each host *h* in hostList **do**

6:  calculate utilization *u* of *h*

7:  **if** *u* >*T*_*u*_ **then**

8:   select VM *v* for migration

9:   add *v* to *M*_*l*_

10:  **end if**

11:  **if** *u* <*T*_*l*_ **then**

12:   **if** *M*_*l*_ is not empty **then**

13:    pick VM *v* from *M*_*l*_

14:    Requested resources *R*_*r*_= resources requested by *v*

15:    supply *s*= allocatable resources of *h*

16:    **if** *R*_*r*_ <*s* **then**

17:     allocate *v* to *h*

18:    **else**

19:     add VMs of *h* to *M*_*l*_

20:     put *h* into sleeping mode

21:    **end if**

22:   **else**

23:    add VMs of *h* to *M*_*l*_

24:    put *h* into sleeping mode

25:   **end if**

26:  **end if**

27:  **if** *u*>*T*_*l*_ & <*T*_*u*_ **then**

28:   **if** *M*_*l*_ is not empty **then**

29:    pick VM *v* from *M*_*l*_

30:    Requested resources *R*_*r*_= resources requested by *v*

31:    calculate utilization *u* of *h*

32:    **if** *u* <*m* **then**

33:     pick VM *v* from *M*_*l*_

34:     **if** *u*+*R*_*r*_ <*T*_*u*_ **then**

35:      allocate *v* to *h*

36:     **else**

37:      check next VM from *M*_*l*_

38:     **end if**

39:    **end if**

40:   **end if**

41:  **end if**

42: **end for**

To find an optimal solution to the problem the proposed algorithm uses GA. The initial population consists of candidate solutions to problem. In case of VMs placement, the solution is described as the mapping of VMs to hosts. A list of hosts is considered as chromosomes and the mappings of VMs to a host are considered as genes. Available hosts are considered in the initial population and the VMs are assigned as per the application demands. The population is created randomly, and the solution is tested to find a better solution. The fitness of the solution is valued based on energy consumption and SLA violations as bi-objective optimization problem. The parameters are calculated with Eqs 16 and 17 respectively. The solution that results in low energy consumption and the minimum number of SLA violations is considered as better solution. Eq 18 [40, 41] is used to calculate the fitness value. Crossover operator is used to produce new solutions by taking genes from two parent solutions. Parents are selected based on tournament selection. Crossover can be either single-point, multi-point, uniform, or ordered crossover. In the proposed algorithm single-point crossover is used. In mutation operator, positions of genes are swapped within a single solution. The positions are swapped based on mutation probability. A random selection occurs based on the probability. In the evaluation of the proposed algorithm crossover and mutation probabilities were set to 0.8 and 0.2 respectively. The pseudo-code of the GA used in this article is shown in Algorithm 2.

**Algorithm 2** Genetic Algorithm

1: **Input**: hostsList

2: **Output**: VMs to hosts placement plan

3: population P=random assignment of VMs to hosts

4: **while** number of iterations left **do**

5:  Evaluate

6:  Crossover

7:  Mutation

8: **end while**

9: return best VMs to hosts placement

## Experimental evaluation

This section presents experimental evaluation of the proposed algorithm. The proposed algorithm was compared with heuristics-based [25] approach and utility-based approach [12]. The heuristics-based approach is used to make a baseline for comparison. The utility-based approach is a specialized model designed to solve the same problem. The main objective of the proposed model is to reduce energy consumption. Different parameters are related to each other. Improving one parameter affects other parameters. To validate the performance of the proposed model QoS parameters including the number of migrations, and the number of host shutdowns are considered SLAVs and included in the evaluation metrics. Algorithms were evaluated based on energy consumption and SLA violations. Energy consumption is calculated as the energy consumed by all hosts as shown in Eq 16. The target is to reduce overall energy consumption. SLA violations occur due to over utilization of hosts or large number of VM migrations. The target is to minimize violations. SLAVs are calcualted using Eq 17. Experiments were performed in CloudSim simulator. Two different types of hosts were used in the simulations. Types include HP ProLiant ML110 G4 and HP ProLiant ML110 G5 as used in previous studies [12, 42]. The data center consists of 100 hosts. VMs with different specifications were included in the experiments. each VM uses one CPU core, 500, 1000, 1500, 2000 MIPS, 1 GB RAM and 10 GB storage. The algorithms were evaluated with tasks of variable workloads as shown in Table 1 [43].

**Table 1:** Tasks types with different parameters.

Task Type	CPU (GHz)	Memory (GBs)	Execution Time ((10^6^*s*)
1	0.02–0.10	0.002–0.015	0.65–1.05
2	0.01–0.04	0.03–0.09	1.60–2.10
3	0.08–0.10	0.002–0.008	0.95–1.40
4	0.02–0.03	0.009–0.13	0.25–0.5
5	0.16–0.17	0.02–0.12	1.65–2.15

**Fig 2:**
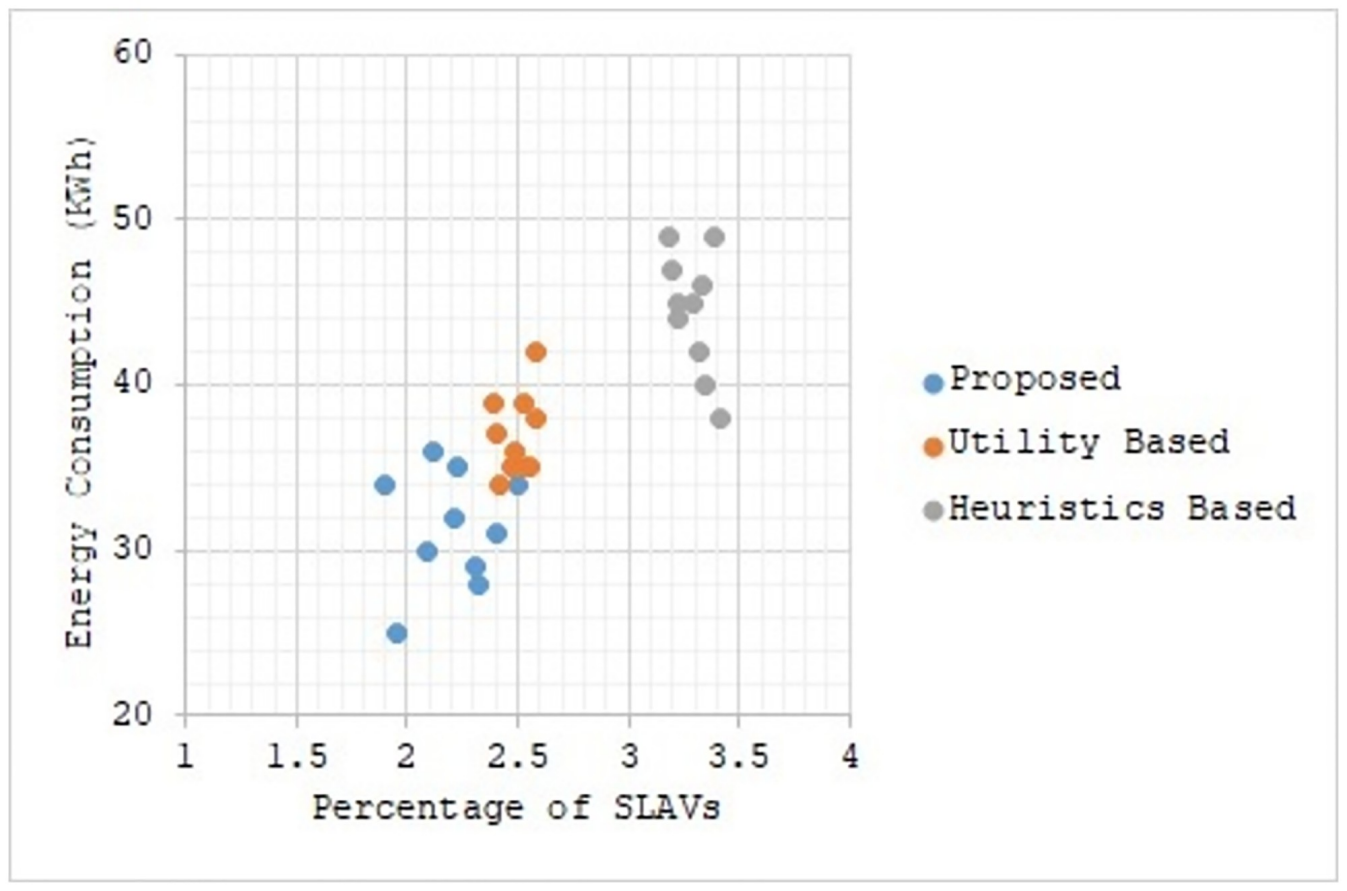
Energy consumption and percentage of times of SLAVs on 50 VMs.

**Fig 3:**
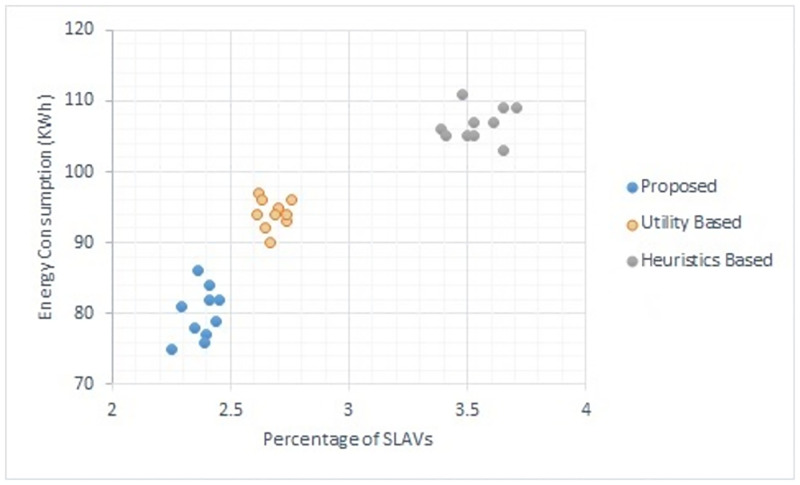
Energy consumption and percentage of times of SLAVs on 100 VMs.

**Fig 4:**
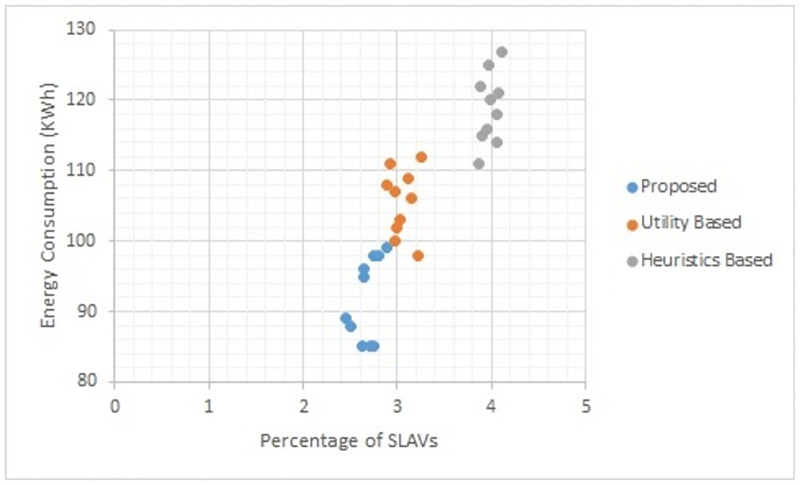
Energy consumption and percentage of times of SLAVs on 150 VMs.

**Fig 5:**
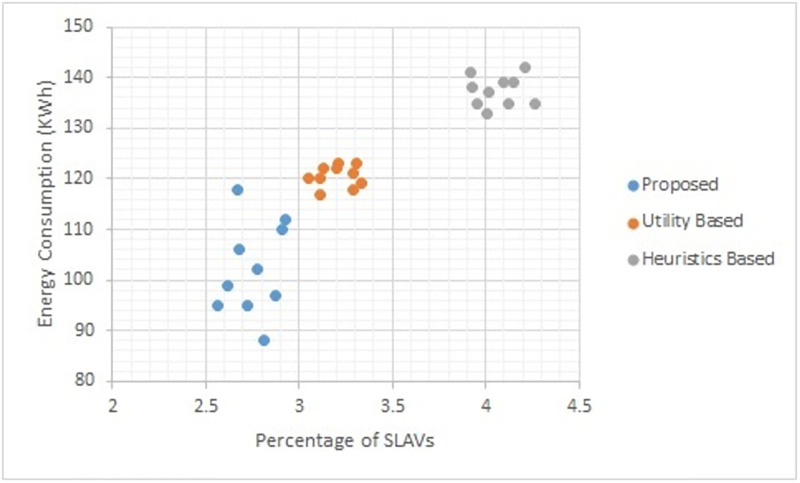
Energy consumption and percentage of times of SLAVs on 200 VMs.

**Fig 6:**
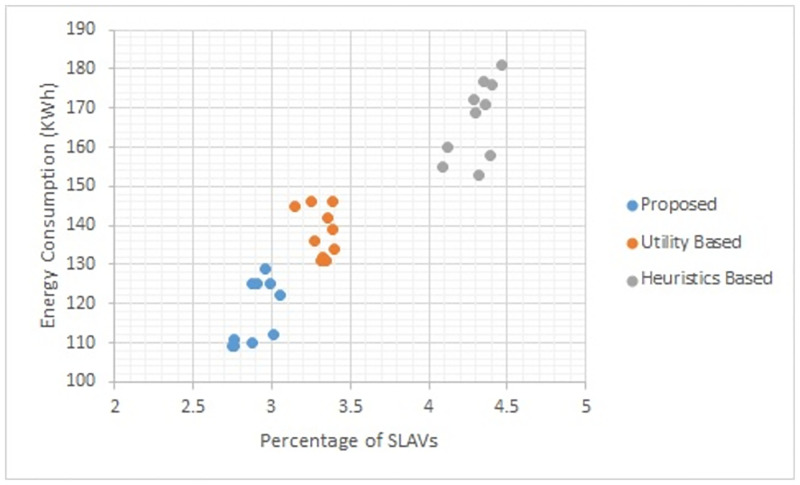
Energy consumption and percentage of times of SLAVs on 250 VMs.

**Fig 7:**
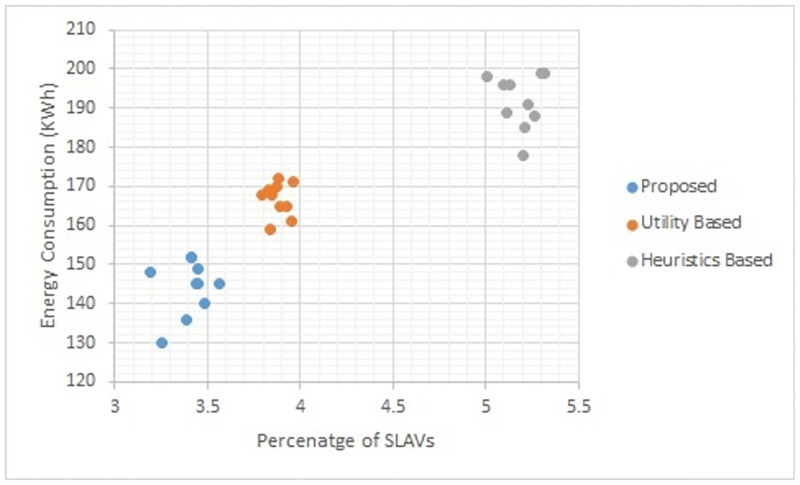
Energy consumption and percentage of times of SLAVs on 300 VMs.

**Fig 8:**
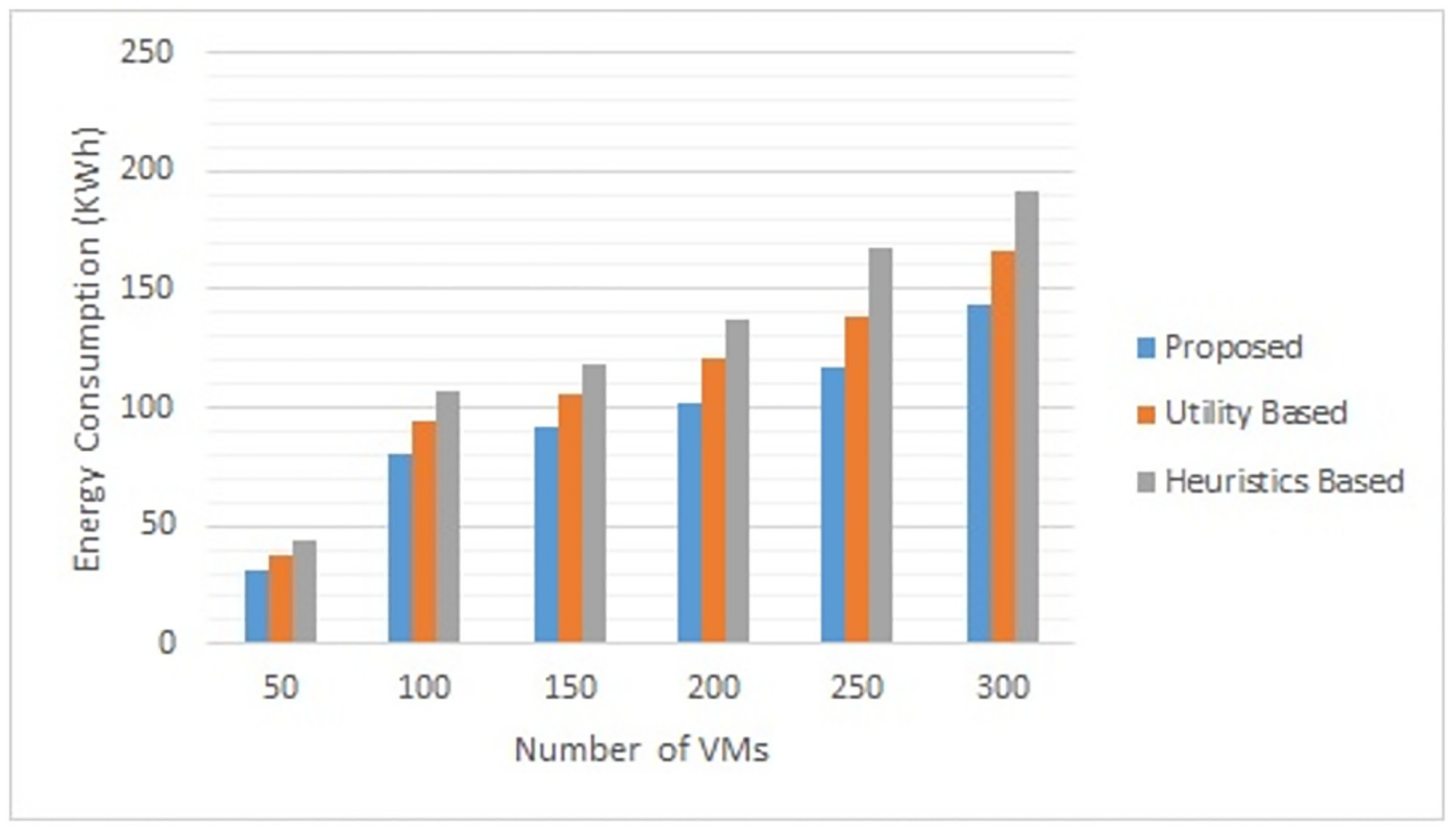
Comparative results of the proposed algorithm with other algorithms for different number of VMs fo energy consumption.

**Fig 9:**
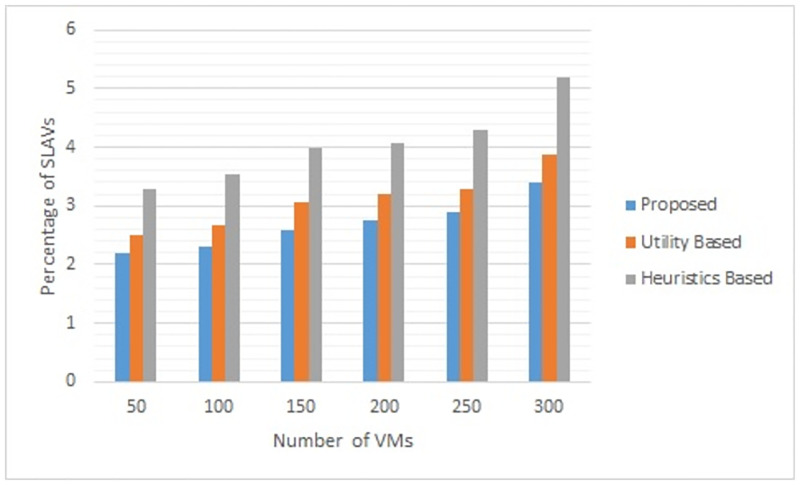
Comparative results of the proposed algorithm with other algorithms for different number of VMs for SLAVs.

Figs 2 to 7 show the results of different configurations of 10 runs of each algorithm. Configurations include different numbers of VMs i.e. 50, 100, 150, 200, 250, 300. Number of hosts for each configuration was 100. Energy consumption and the percentage of SLAVs are plotted for all algorithms. The results show that the proposed algorithm reduced both energy consumption and SLAVs as compared to other algorithms. In case of 50 VMs, the proposed algorithm achieved 15.13 and 28.63% reduction in energy consumption in comparison to utility-based approach and heuristics-based approach respectively. Reduction in SLAVs in comparison to utility based and heuristics-based approaches is 11.67 and 33.14% respectively for the same configuration. For 100 VMs the percentage improvement of the proposed algorithm over the compared algorithms is 14.98, 25.02% for energy consumption and 11.41, 33.02% for SLAVs. In case of 150 VMs, the percent improvement gain of the proposed algorithm over the utility-based approach is 13.06% for energy consumption and 12.35% for SLAVs. For the same configuration, the percent improvement gain of the proposed algorithm over heuristics-based approach is 22.79% for energy consumption and 32.86% for SLAVs. For 200 VMs and 100 hosts, the proposed algorithm achieved percent improvement gain of 15.18 and 13.98% over utility-based approach for energy consumption and SLAVs respectively. The improvement over the heuristics-based approach for the same configuration is 25.61 and 32.25% for energy consumption and SLAVs respectively. In case of 250 VMs, the improvement of the proposed algorithm over utility-based and heuristics-based approaches for energy consumption is 14.83 and 29.6% whereas for SLAVs the improvement is 12.8 and 32.82% respectively. For 300 VMs the proposed algorithm achieved 13.45 and 12.27% improvement for energy consumption and SLAVs respectively. For the same configuration, the improvement over heuristics-bases approach is 24.85 and 34.39% for energy consumption and SLAVs respectively. Figs 8 and 9 show average comparative results of energy consumption and SLAVs respectively. It can be observed that as the number of VMs increases, both energy consumption and SLAVs also increase. Energy consumption increases as more hosts are needed to accommodate VMs. SLAVs increase because more requests for VMs placement to host cause over-utilization and VMs migrations also take place. The proposed algorithm uses adaptive thresholds to identify overload and under-load hosts. Additionally, a third value is used to identify hosts that can accommodate VMs while the load remains normal. This leads to efficient resource utilization and both energy consumption and SLVs are reduced. In addition, the proposed algorithm assigns tasks to VMs according to the demand and capacity which ensures QoS and reduces SLAVS.

## Conclusion

In this paper, a novel algorithm for VMs placement in cloud computing environment is presented. The algorithm uses adaptive thresholds to identify overutilized and underutilized hosts. GA is used for optimization. The algorithm targets energy consumption, and SLAVs as bi-objective parameters. A comparative analysis of the experimental results is presented to validate the proposed algorithm. Many studies have focused on some set of parameters, while others are not considered. The developed methods should consider objectives that are related to each other. Improving one objective affects other objectives and makes it difficult to implement. Cloud computing is vulnerable to various security attacks and the developed methods should consider this objective as well.

## Supporting information

S1 Dataset (PDF)

## References

[1] Bharathi PD, Prakash P, Kiran MVK. Virtual machine placement strategies in cloud computing. In: 2017 Innovations in Power and Advanced Computing Technologies (i-PACT). IEEE; 2017. p. 1–7.

[2] Muteeh A, Sardaraz M, Tahir M. MrLBA: multi-resource load balancing algorithm for cloud computing using ant colony optimization. Cluster Computing. 2021;24(4):3135–3145. doi: 10.1007/s10586-021-03322-3

[3] Dashti SE, Rahmani AM. Dynamic VMs placement for energy efficiency by PSO in cloud computing. Journal of Experimental & Theoretical Artificial Intelligence. 2016;28(1-2):97–112. doi: 10.1080/0952813X.2015.1020519

[4] Choudhary A, Rana S, Matahai K. A critical analysis of energy efficient virtual machine placement techniques and its optimization in a cloud computing environment. Procedia Computer Science. 2016;78:132–138. doi: 10.1016/j.procs.2016.02.022

[5] Liu L, Zhang M, Lin Y, Qin L. A survey on workflow management and scheduling in cloud computing. In: 2014 14th IEEE/ACM International Symposium on Cluster, Cloud and Grid Computing. IEEE; 2014. p. 837–846.

[6] Masdari M, Salehi F, Jalali M, Bidaki M. A survey of PSO-based scheduling algorithms in cloud computing. Journal of Network and Systems Management. 2017;25(1):122–158. doi: 10.1007/s10922-016-9385-9

[7] Masdari M, ValiKardan S, Shahi Z, Azar SI. Towards workflow scheduling in cloud computing: a comprehensive analysis. Journal of Network and Computer Applications. 2016;66:64–82. doi: 10.1016/j.jnca.2016.01.018

[8] Zhang Q, Cheng L, Boutaba R. Cloud computing: state-of-the-art and research challenges. Journal of internet services and applications. 2010;1(1):7–18. doi: 10.1007/s13174-010-0007-6

[9] M M, Ali AM. Sustainable Supply Chain Management in the Age of Machine Intelligence: Addressing Challenges, Capitalizing on Opportunities, and Shaping the Future Landscape. Sustainable MAchine Intelligence. 2023;3:1–14.

[10] Karam M Sallam AWM, Mohamed M. Internet of Things (IoT) in Supply Chain Management: Challenges, Opportunities, and Best Practices. Sustainable MAchine Intelligence. 2023;2:12–44.

[11] Nabeeh NA. Assessment and Contrast the Sustainable Growth of Various Road Transport Systems using Intelligent Neutrosophic Multi-Criteria Decision-Making Model. Sustainable MAchine Intelligence. 2023;2:1–12.

[12] Mosa A, Paton NW. Optimizing virtual machine placement for energy and SLA in clouds using utility functions. Journal of Cloud Computing. 2016;5(1):17. doi: 10.1186/s13677-016-0067-7

[13] Yu Y, Gao Y. Constraint programming-based virtual machines placement algorithm in datacenter. In: International Conference on Intelligent Information Processing. Springer; 2012. p. 295–304.

[14] Dörterler S, Dörterler M, Ozdemir S. Multi-objective virtual machine placement optimization for cloud computing. In: 2017 International Symposium on Networks, Computers and Communications (ISNCC). IEEE; 2017. p. 1–6.

[15] Li K, Nabrzyski J. Virtual machine placement in cloudlet mesh with network topology reconfigurability. In: 2017 IEEE 6th International Conference on Cloud Networking (CloudNet). IEEE; 2017. p. 1–7.

[16] López-Pires F, Barán B, Benítez L, Zalimben S, Amarilla A. Virtual machine placement for elastic infrastructures in overbooked cloud computing datacenters under uncertainty. Future Generation Computer Systems. 2018;79:830–848. doi: 10.1016/j.future.2017.09.021

[17] Maryam K, Sardaraz M, Tahir M. Evolutionary Algorithms in Cloud Computing from the Perspective of Energy Consumption: A Review. In: 2018 14th International Conference on Emerging Technologies (ICET). IEEE; 2018. p. 1–6.

[18] Wang W, Zhao Y, Tornatore M, Gupta A, Zhang J, Mukherjee B. Virtual machine placement and workload assignment for mobile edge computing. In: 2017 IEEE 6th International Conference on Cloud Networking (CloudNet). IEEE; 2017. p. 1–6.

[19] Kumar T S, Mustapha SDS, Gupta P, Tripathi RP. Hybrid approach for resource allocation in cloud infrastructure using random forest and genetic algorithm. Scientific Programming. 2021;2021:1–10.

[20] Dong YS, Xu GC, Fu XD. A distributed parallel genetic algorithm of placement strategy for virtual machines deployment on cloud platform. The Scientific World Journal. 2014;2014. doi: 10.1155/2014/259139 25097872 PMC4109368

[21] Liu B, Chen R, Lin W, Wu W, Lin J, Li K. Thermal-aware virtual machine placement based on multi-objective optimization. The Journal of Supercomputing. 2023; p. 1–28.

[22] Li Z, Pan M, Yu L. Multi-resource collaborative optimization for adaptive virtual machine placement. PeerJ Computer Science. 2022;8:e852. doi: 10.7717/peerj-cs.852 35111927 PMC8771770

[23] Yang S, Wieder P, Yahyapour R, Trajanovski S, Fu X. Reliable virtual machine placement and routing in clouds. IEEE Transactions on Parallel and Distributed Systems. 2017;28(10):2965–2978. doi: 10.1109/TPDS.2017.2693273

[24] Thabet M, Hnich B, Berrima M. A sampling-based online Co-Location-Resistant Virtual Machine placement strategy. Journal of Systems and Software. 2022;187:111215. doi: 10.1016/j.jss.2022.111215

[25] Beloglazov A, Abawajy J, Buyya R. Energy-aware resource allocation heuristics for efficient management of data centers for cloud computing. Future generation computer systems. 2012;28(5):755–768. doi: 10.1016/j.future.2011.04.017

[26] Zhou A, Wang S, Cheng B, Zheng Z, Yang F, Chang RN, et al. Cloud service reliability enhancement via virtual machine placement optimization. IEEE Transactions on Services Computing. 2017;10(6):902–913. doi: 10.1109/TSC.2016.2519898

[27] Hormozi E, Hu S, Ding Z, Tian YC, Wang YG, Yu ZG, et al. Energy-efficient virtual machine placement in data centres via an accelerated Genetic Algorithm with improved fitness computation. Energy. 2022;252:123884. doi: 10.1016/j.energy.2022.123884

[28] Jian C, Bao L, Zhang M. A high-efficiency learning model for virtual machine placement in mobile edge computing. Cluster Computing. 2022;25(5):3051–3066. doi: 10.1007/s10586-022-03550-1

[29] Peake J, Amos M, Costen N, Masala G, Lloyd H. PACO-VMP: parallel ant colony optimization for virtual machine placement. Future Generation Computer Systems. 2022;129:174–186. doi: 10.1016/j.future.2021.11.019

[30] Li L, Liu K. Guarantee-aware cost effective virtual machine placement algorithm for the cloud. In: 2017 IEEE 19th International Conference on High Performance Computing and Communications; IEEE 15th International Conference on Smart City; IEEE 3rd International Conference on Data Science and Systems (HPCC/SmartCity/DSS). IEEE; 2017. p. 506–513.

[31] Liang X, Gui X, Jian A, Ren D. Mitigating cloud co-resident attacks via grouping-based virtual machine placement strategy. In: 2017 IEEE 36th International Performance Computing and Communications Conference (IPCCC). IEEE; 2017. p. 1–8.

[32] Liu XF, Zhan ZH, Deng JD, Li Y, Gu T, Zhang J. An energy efficient ant colony system for virtual machine placement in cloud computing. IEEE transactions on evolutionary computation. 2018;22(1):113–128. doi: 10.1109/TEVC.2016.2623803

[33] Zhao H, Wang J, Liu F, Wang Q, Zhang W, Zheng Q. Power-aware and performance-guaranteed virtual machine placement in the cloud. IEEE Transactions on Parallel and Distributed Systems. 2018;29(6):1385–1400. doi: 10.1109/TPDS.2018.2794369

[34] Sonklin C, Tang M, Tian YC. A decrease-and-conquer genetic algorithm for energy efficient virtual machine placement in data centers. In: 2017 IEEE 15th International Conference on Industrial Informatics (INDIN). IEEE; 2017. p. 135–140.

[35] Jiang C, Wu J, Li Z. Adaptive thresholds determination for saving cloud energy using three-way decisions. Cluster Computing. 2018; p. 1–8.

[36] Marahatta A, Pirbhulal S, Zhang F, Parizi RM, Choo KKR, Liu Z. Classification-based and Energy-Efficient Dynamic Task Scheduling Scheme for Virtualized Cloud Data Center. IEEE Transactions on Cloud Computing. 2019;.

[37] Malik N, Sardaraz M, Tahir M, Shah B, Ali G, Moreira F. Energy-efficient load balancing algorithm for workflow scheduling in cloud data centers using queuing and thresholds. Applied Sciences. 2021;11(13):5849. doi: 10.3390/app11135849

[38] Monil MAH, Qasim R, Rahman RM. Energy-aware VM consolidation approach using combination of heuristics and migration control. In: Ninth International Conference on Digital Information Management (ICDIM 2014). IEEE; 2014. p. 74–79.

[39] Casas I, Taheri J, Ranjan R, Wang L, Zomaya AY. GA-ETI: An enhanced genetic algorithm for the scheduling of scientific workflows in cloud environments. Journal of computational science. 2018;26:318–331. doi: 10.1016/j.jocs.2016.08.007

[40] Sardaraz M, Tahir M. A Hybrid Algorithm for Scheduling Scientific Workflows in Cloud Computing. IEEE Access. 2019;7(1):186137–186146. doi: 10.1109/ACCESS.2019.2961106

[41] Sardaraz M, Tahir M. A parallel multi-objective genetic algorithm for scheduling scientific workflows in cloud computing. International Journal of Distributed Sensor Networks. 2020;16(8):1550147720949142. doi: 10.1177/1550147720949142

[42] Beloglazov A, Buyya R. Optimal online deterministic algorithms and adaptive heuristics for energy and performance efficient dynamic consolidation of virtual machines in cloud data centers. Concurrency and Computation: Practice and Experience. 2012;24(13):1397–1420. doi: 10.1002/cpe.1867

[43] Marahatta A, Wang Y, Zhang F, Sangaiah AK, Tyagi SKS, Liu Z. Energy-aware fault-tolerant dynamic task scheduling scheme for virtualized cloud data centers. Mobile Networks and Applications. 2019;24:1063–1077. doi: 10.1007/s11036-018-1062-7

