# Supplementary material for: Energy efficient virtual machines placement in cloud datacenters using genetic algorithm and adaptive thresholds
Source: PLoS One. 2024 Jan 2;19(1):e0296399. doi: 10.1371/journal.pone.0296399 (PMC10760894; doi:10.1371/journal.pone.0296399)
Supplement: S1 Dataset — (PDF) [file pone.0296399.s001.pdf]

Energy Efficient Virtual Machines Placement in Cloud Datacenters Using Genetic Algorithm and Adaptive Thresholds.

Supplementary Table S1. Types of tasks used to evaluate the proposed framework.

Task Type	CPU (GHz)	Memory (GBs)	Execution Time ((10^6 s)
1	0.02-0.10	0.002-0.015	0.65-1.05
2	0.01-0.04	0.03-0.09	1.60-2.10
3	0.08-0.10	0.002-0.008	0.95-1.40
4	0.02-0.03	0.009-0.13	0.25-0.5
5	0.16-0.17	0.02-0.12	1.65-2.15
